# Risk of intraoperative floppy iris syndrome among selective alpha-1 blockers—A consistency model of 6,488 cases

**DOI:** 10.3389/fmed.2022.941130

**Published:** 2022-08-30

**Authors:** Ya-Hui Wang, Liang-Chen Huang, Sung Huang Laurent Tsai, Ying-Jen Chen, Chien-Liang Wu, Yi-No Kang

**Affiliations:** ^1^Department of Ophthalmology, Taipei Municipal Wanfang Hospital, Taipei, Taiwan; ^2^School of Medicine, Taipei Medical University, Taipei, Taiwan; ^3^Division of Urology, Department of Surgery, En Chu Kong Hospital, New Taipei City, Taiwan; ^4^Department of Orthopaedic Surgery, Chang Gung Memorial Hospital, Keelung, Taiwan; ^5^School of Medicine, Chang Gung University, Taoyuan, Taiwan; ^6^Department of Geriatric and General Internal Medicine Chang Gung Memorial Hospital, Taoyuan, Taiwan; ^7^Evidence-Based Medicine Center, Wan Fang Hospital, Taipei Medical University, Taipei, Taiwan; ^8^Research Center of Big Data and Meta-Analysis, Wan Fang Hospital, Taipei Medical University, Taipei, Taiwan; ^9^Cochrane Taiwan, Taipei Medical University, Taipei, Taiwan; ^10^Institute of Health Policy and Management, College of Public Health, National Taiwan University, Taipei, Taiwan; ^11^Department of Health Care Management, College of Health Technology, National Taipei University of Nursing Health Sciences, Taipei, Taiwan

**Keywords:** phacoemulsification surgery, cataract surgery, intraoperative floppy iris syndrome, prostate hyperplasia, α1-antagonists, tamsulosin, doxazosin, silodosin

## Abstract

Selective α1-blockers are commonly administered to patients with lower urinary tract syndrome and benign prostatic hyperplasia, but may increase the risk of intraoperative floppy iris syndrome (IFIS). The purpose of this study aimed to clarify the risk of IFIS among various selective α1-blockers. Four databases were searched for prospective studies comparing alpha-1-antagonists. Data were pooled using the consistency model, and used risk ratio (RR) and mean difference (MD) for IFIS and pupil diameter, respectively. This study finally included 25 prospective comparative studies. Based on 51 direct comparisons with 6488 cases, risks of IFIS in patients who received tamsulosin [RR, 13.85; 95% confidence interval (CI): 7.34 to 26.11], terazosin (RR, 8.94; 95% CI 2.88 to 27.74), alfuzosin (RR, 7.73; 95% CI: 3.05 to 19.62), and doxazosin (RR, 3.88; 95% CI: 1.13 to 13.28) were significantly higher than those did not receive α1-antagonists. Based on 11 direct comparisons with 564 cases, as compared to no α1-antagonists, patients who received tamsulosin (MD, −0.36; 95% CI: −0.71 to −0.01) and alfuzosin (MD, −0.34; 95% CI: −0.62 to −0.07) showed smaller pupil diameter under mesopic light levels, while those received silodosin did not show significantly smaller mesopic pupil diameter than people without α1-antagonists. IFIS seems to be inevitable with the usage of α1-antagonists, and tamsulosin needs to be cautious due to the significantly higher risk of severe IFIS. With regard to silodosin, there is no strong evidence to support the uses of italthough it does not significantly decrease mesopic pupil diameter.

## Introduction

Lower urinary tract syndrome (LUTS) and benign prostatic hyperplasia (BPH) affect the overall quality of life in a large population in every country (1), which are linked to several complications (2–7). BPH/LUTS can be treated pharmacologically and surgically. Medication armamentarium against BPH/LUTS included α1-adrenergic antagonists, 5α-reductase inhibitors, antimuscarinics, phosphodiesterase type 5 inhibitors, β3-agonists, and numerous plant extracts. α1-antagonists are usually the first-line treatment of BPH/LUTS in men (8, 9). Cardiovascular adverse effects (postural hypotension, syncope, vertigo, and dizziness) and CNS adverse effects (somnolence, asthenia) from α1-adrenergic blockade led to falls, fractures, and institutionalization in elders; ejaculation disorder was also associated with these medications (10). Selective α1-antagonists (such as terazosin, and doxazosin) and uroselective α1-antagonists (tamsulosin, alfuzosin, silodosin, and naftopidil) then sprang up with higher selectivity to α1-a or α1-d adrenergic receptors instead of α1-b receptors.

One notorious yet often omitted complication bound with α1-antagonists was intraoperative floppy iris syndrome (IFIS), which was firstly introduced in patients under tamsulosin in 2005 (11). Not only IFIS was influenced by α1-antagonist in the ophthalmological field; others were to pupil, choroid, and iris (12, 13), which obstructed one from clear vision and well-being. Unfortunately, most the male patients with BPH/LUTS are candidates for cataract and phacoemulsification surgery (PCS). The prevalence of BPH/LUTS and cataracts both increased with age, which was 50 and 3.9% around the sixth decade, respectively, while they increased up to 80 and 92.6% at age over 80 (14, 15).

IFIS mainly occurs during cataract surgery and is defined into four grades based on the signs observed intraoperatively: (1) no IFIS, mild, moderate, and severe IFIS. No IFIS refers to stable and normal iris without significant miosis; (2) mild IFIS stands for slightly noticeable floppy iris with minor or no miosis but no tendency of iris prolapse; (3) moderate IFIS floppy iris means significant miosis and small tendency toward iris prolapse; and (4) severe IFIS is floppy iris with significant miosis and a strong tendency toward iris prolapse (16). The occurrence of unanticipated IFIS is accompanied by increased rates of multiple intraoperative complications, including corneal endothelial loss, iris injury, anterior capsule tears, posterior capsule rupture, vitreous loss, retained nuclear fragments, as well as postoperative complications, including intraocular pressure elevation, cystoid macular edema and postoperative ocular inflammation (11, 17, 18). These features make IFIS an important issue for ophthalmologists to prevent and manage appropriately.

Choosing α1-antagonists for male patients with BPH/LUTS, therefore, is a vital issue. Evidence regarding IFIS or pupil diameter after selective α1-antagonists for patients with BPH/LUTS disperse in many studies with varying findings based on different selective α1-antagonists. However, few syntheses have provided quantitative evidence on this topic concurrently covering various selective α1-antagonists. To fill up the paucity, the present study proposed a network meta-analysis of prospective comparative studies because a consistency model would be a methodological solution for pooling data of IFIS and pupil diameter after various α1-antagonists in patients with BPH/LUTS. The purpose of this network meta-analysis was to clarify the risk among commonly used α1-antagonists against urological problems and their influence on the ophthalmological field through a testing risk of IFIS and pupil diameter after selective α1-antagonists for patients with BPH/LUTS. The research question has been structured in PICO format as follows:

**Patient**: patients with BPH/LUTS

**Intervention**: selective α1-antagonists (e.g. terazosin, doxazosin, tamsulosin, alfuzosin, silodosin, and naftopidil)

**Control**: without selective α1-antagonists

**Outcome**: IFIS and pupil diameter.

## Methods

To obtain reliable findings, the present synthesis was to pool data from studies that met the following criteria: (a) study with a prospective comparative design, (b) all subjects with BPH/LUTS, (c) investigation of selective α1-antagonists exposure before measurements of IFIS or pupil diameter. The exclusion criteria were as follows: (a) study without human subjects, (b) recruitment of both BPH/LUTS and non-BPH/LUTS cases, (c) study without a clear definition of exposure of selective α1-antagonists with separation of each selective α1-antagonists, (d) study without clear description results of IFIS or pupil diameter evaluation, (e) study without any comparative group. The protocol of this synthesis has been registered on the PROSPERO prior to the start of this study, and the protocol number is CRD42020191759.

### Data sources and evidence selection

Comprehensive searches of four electronic databases (PubMed database of the National Library of Medicine, EMBASE, Cochrane CENTRAL, and Web of Science) from their inceptions were performed (with no language restrictions), and hand search reference lists were done up to November 2021. The search consisted of three parts in terms of population with BPH/LUTS, exposure of selective α1-blockers, and relevant situation of eye conditions. Consequently, keywords for the target population were prostatic hyperplasia, urinary (a sensitive word instead of LUTS), as well as void. Keywords for α1-antagonists covered alpha antagonist, alpha-blocker, terazosin, tamsulosin, alfuzosin, silodosin, naftopidil, and doxazosin. Keywords for the relevance of ophthalmology in this topic were cataract, phacoemulsification, floppy iris, lens, iris, cornea, choroid, pupil, and ophthalmology. The search concurrently used both free-texts and medical subject headings (MeSH) of these three parts of keywords. The Boolean operator OR was applied to take a union of keywords in each search part, and then the Boolean operator AND was further applied to take the intersection among the three parts. Supplementary Table S1 details an example of the search process.

The titles and abstracts were screened for possible inclusion. Our team members (Dr. L-C Huang and Dr. Y-H. Wang) also examined all references of relevant reviews and eligible articles that our search retrieved. Then, the two members independently reviewed all titles and abstracts. Articles were selected for full-text review if inclusion criteria are met according to either reviewer, with a low threshold for retrieval. Disagreements were resolved by discussion in team meeting in which an experienced researcher participated in the determination of evidence selection.

### Data extraction and quality evaluation

The two team members further independently extracted information using a piloted data collection form after evidence selection. The following information was planned to be extracted general study characteristics, potential effect modifiers (age and sex), and outcome data. With regard to general study characteristics, they tried to extract study origin (country), study setting, inclusion and exclusion criteria, and description of the exposure and comparator (timing of administration, dose, method of administration, duration of exposure). Concerning outcome data, the two members extracted number of total IFIS (and also severe IFIS) events with the total number of participants in both α1-blocker exposure and control groups; and they also extracted means, standard deviations, and group size for pupil diameter. Due to various conditions for measurement of pupil diameter, our team distinguished data of pupil diameter from two conditions, including under mesopic light levels and after dilation.

On the basis of the work of information extraction, the two members independently evaluated the risk of bias within each study included in the present synthesis. Due to prospective comparative design, they critically appraised the studies using Newcastle-Ottawa Quality Assessment Scale, in which bias of selection, comparability, and outcome assessment were evaluated. When their evaluation differed, the disagreements were also resolved by discussion in team meeting with an experienced researcher for determining the quality evaluation.

### Data synthesis and analysis

To obtain an overview of the risk of IFIS and the difference in pupil diameter among selective α1-blockers, this study conducted a quantitative synthesis using network meta-analysis based on frequentist approach. In other words, this synthesis pooled direct and indirect evidence in a consistency model. Outcomes in the present study were primarily incidence of IFIS (binary variable) and pupil diameter (continuous variable); wherefore different statistical measurements were used. Risk ratio (RR) was used to present pooled results of overall IFIS and severe IFIS. On the other hand, due to continuous data on pupil diameter, mean difference (MD) was used for the pooled comparisons of the diameters among selective α1-blockers and control groups. For the determination of statistical significance and precision in each analysis, 95% confidence interval (CI) was calculated.

To evaluate the quality of the consistency model of IFIS and pupil diameter, this study tested global incoherence between direct and indirect evidence. Because the present network meta-analysis was contributed by two-group, three-group, four-group, and five-group studies, incoherence tests were based on the design-by-treatment interaction model. As the test of global incoherence reached statistical significance, the present study further tested local incoherence using the side-splitting method. In addition to the incoherence test, this study also examined small-study effects using the comparison-adjusted funnel plot with Egger's regression intercept test when there were around 10 comparisons in a consistency model. *P*-scores were presented to help readers determine the most optimal selective α1-blocker. The abovementioned analyses were carried out using R version 4.1.0 with packages “meta” (5.1-1) and “netmeta” (2.0-1) via RStudio version 1.4.1717.

## Results

**Figure 1:**
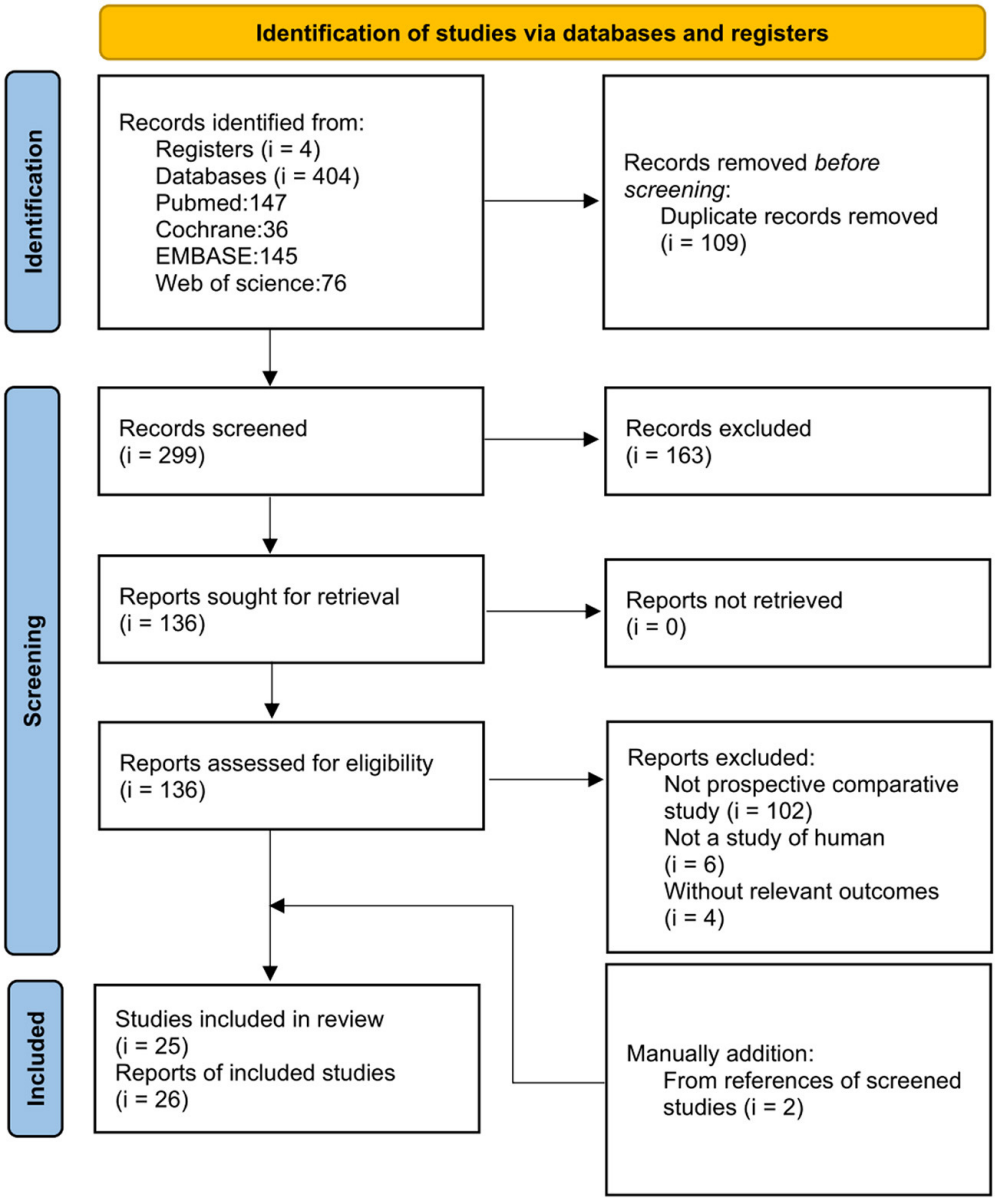
Flowchart of evidence selection for the synthesis of the usage of α1-antagonists in patients with lower urinary tract syndrome or benign prostatic hyperplasia.

A total of 410 references were found after database search (*i* = 408) and manually reference check (*i* = 2). Twenty-six references based on 25 studies reported outcomes of IFIS or pupil diameter after using selective α1-blockers among patients with BPH or LUTS in prospective comparative studies and were included in the present synthesis (12, 13, 16, 19–41). These 25 studies covered most selective α1-blockers for patients with BPH or LUTS. However, no documented IFIS with naftopidil was found in this comprehensive search. Figure 1 shows the process of evidence selection.

### Characteristics and quality of included studies

Though a comprehensive search, this study identified 25 prospective comparative studies covering five selective α1-blockers, including tamsulosin, alfuzosin, doxazosin, terazosin, and silodosin. These comparative studies prospectively recruited patients from Denmark, India, Poland, Romania, Spain, Turkey, the UK, and the USA. Publication years covered 2006 and 2021. Available information from the included articles revealed that a total of 16 studies only recruited males, and mean ages ranged from 58 to 79 years old. Table 1 shows relevant information about each study. Based on quality assessment (Supplementary Table S2), half studies seemed to be of good quality (*i* = 13), 20% of studies (*i* = 5) appeared to be fair quality, and the others were of poor quality.

**Table 1:** Characteristics of the included randomized controlled trials.

**Author**	**Country**	**α1-blocker/control**	**Patients (eyes)**	**Sex: Male %**	**Age** **(Mean ± SD)**
Aktaset al. (35)	USA	Tamsulosin	16 (16)	100%	59.8 ± 6.7
		Alfuzosin	14 (14)	100%	60.9 ± 5.8
		Control	18 (18)	100%	62.4 ± 7.1
Altanet al. (19)	Turkey	Tamsulosin	32 (64)	100%	61.0 ± 8.1
		Alfuzosin	32 (64)	100%	58.7 ± 9.6
Bidagurenet al. (20)	Spain	Tamsulosin	19 (19)	100%	76.7 ± 5.81
		Control	19 (19)	100%	75.3 ± 4.77
Casuccioet al. (25)	USA	Tamsulosin	50 (50)	100%	75.2 ± 6.2
		Alfuzosin	15 (15)	100%	Unclear
		Doxazosin	20 (20)	100%	Unclear
		Terazosin	15 (15)	100%	Unclear
		Control	50 (50)	100%	73.8 ± 10.5
Chadhaet al. (21)	UK	Tamsulosin	21 (21)	95.24%	Unclear
		Alfuzosin	2 (2)	100%	Unclear
		Doxazosin	48 (50)	37.5%	Unclear
		Terazosin	1 (1)	100%	Unclear
		Control	1,696 (1,772)	Unclear	Unclear
Changet al. (30)	USA	Tamsulosin	Unclear (70)	100%	76.8 ± 7.1
		Alfuzosin	Unclear (43)	100%	75.5 ± 7.0
		Control	Unclear (113)	100%	74.8 ± 9.8
Chatziralliet al. (38)	UK	Tamsulosin	Unclear (135)	Unclear	Unclear
		Alfuzosin	Unclear (121)	Unclear	Unclear
		Terazosin	Unclear (55)	Unclear	Unclear
		Control	Unclear (963)	Unclear	Unclear
Doganet al. (13)	Turkey	Tamsulosin	31 (31)	100%	59.73 ± 10.37
		Alfuzosin	32 (32)	100%	63.43 ± 8.01
Goyalet al. (31)	India	Tamsulosin	41 (Unclear)	Unclear	Unclear
		Alfuzosin	18 (Unclear)	Unclear	Unclear
		Control	944 (Unclear)	Unclear	Unclear
Hargitaiet al. (27)	Denmark	Tamsulosin	30 (30)	100%	78.60 ± 10.35
		Control	31 (31)	100%	78.48 ± 5.84
Hillelsohnet al. (36)	USA	Tamsulosin	38 (38)	Unclear	65 ± 13.0
		Control	43 (43)	Unclear	61.4 ± 13.05
Horvathet al. (26)	Romania	Tamsulosin	15 (15)	Unclear	Unclear
		Control	423 (424)	Unclear	Unclear
Kaczmareket al. (39)	Poland	Tamsulosin	18 (18)	Unclear	Unclear
		Doxazosin	24 (24)	Unclear	Unclear
		Control	277 (277)	Unclear	Unclear
Kanaret al. (40)	Turkey	Tamsulosin	46 (92)	100%	65.2 ± 8.01
		Silodosin	41 (82)	100%	64.87 ± 7.17
Karacaet al. (41)	Turkey	Silodosin	74 (unclear)	100%	63.35 ± 7.21
		Control	30 (unclear)	100%	63.07 ± 4.73
Keklikciet al. (23)	Turkey	Tamsulosin	23 (23)	100%	Unclear
		Control	556 (571)	54.86%	Unclear
Klysiket al. (32)	Poland	Tamsulosin	25 (25)	100%	Unclear
		Alfuzosin	9 (9)	100%	Unclear
		Doxazosin	26 (26)	100%	Unclear
		Terazosin	11 (11)	100%	Unclear
Limet al. (33)	Korea	Tamsulosin	15 (21)	100%	Unclear
		Alfuzosin	2 (3)	100%	Unclear
		Doxazosin	2 (2)	100%	Unclear
		Terazosin	2 (4)	100%	Unclear
		Control	15 (30)	100%	72.32 ± 6.174
Ozeret al. (9)	Turkey	Tamsulosin	5 (5)	100%	77.25 ± 6.3
		Control	421 (421)	51.54%	58 ± 4.3
Theodossiadis (12)	Greece	Tamsulosin	15 (30)	100%	Unclear
		Alfuzosin	22 (44)	100%	Unclear
		Control	25 (50)	100%	Unclear
Storr-Paulsen (34)	Denmark	Tamsulosin	23 (23)	100%	79.9 ± 7.3
		Control	25 (25)	100%	76.7 ± 7.1
Prataet al. (24)	USA	Tamsulosin	27 (43)	100%	Unclear
		Terazosin	2 (2)	100%	Unclear
		Control	22 (31)	100%	67.1 ± 9.1
Takmazet al. (22)	Turkey	Tamsulosin	17 (18)	Unclear	70.2 ± 6.8
		Alfuzosin	2 (2)	Unclear	Unclear
		Terazosin	4 (4)	Unclear	Unclear
		Control	751 (834)	Unclear	Unclear
Tufanet al. (29)	Turkey	Tamsulosin	16 (32)	100%	Unclear
		Alfuzosin	4 (8)	100%	Unclear
		Doxazosin	1 (2)	100%	Unclear
		Terazosin	2 (4)	100%	Unclear
		Control	26 (42)	100%	60.3 ± 8.2
Yukselet al. (37)	Turkey	Tamsulosin	29 (29)	100%	63.9 ± 8.1
		Doxazosin	27 (27)	100%	60.2 ± 6.2
		Control	40 (40)	100%	61.2 ± 8.5

### Intraoperative floppy iris syndrome

**Figure 2:**
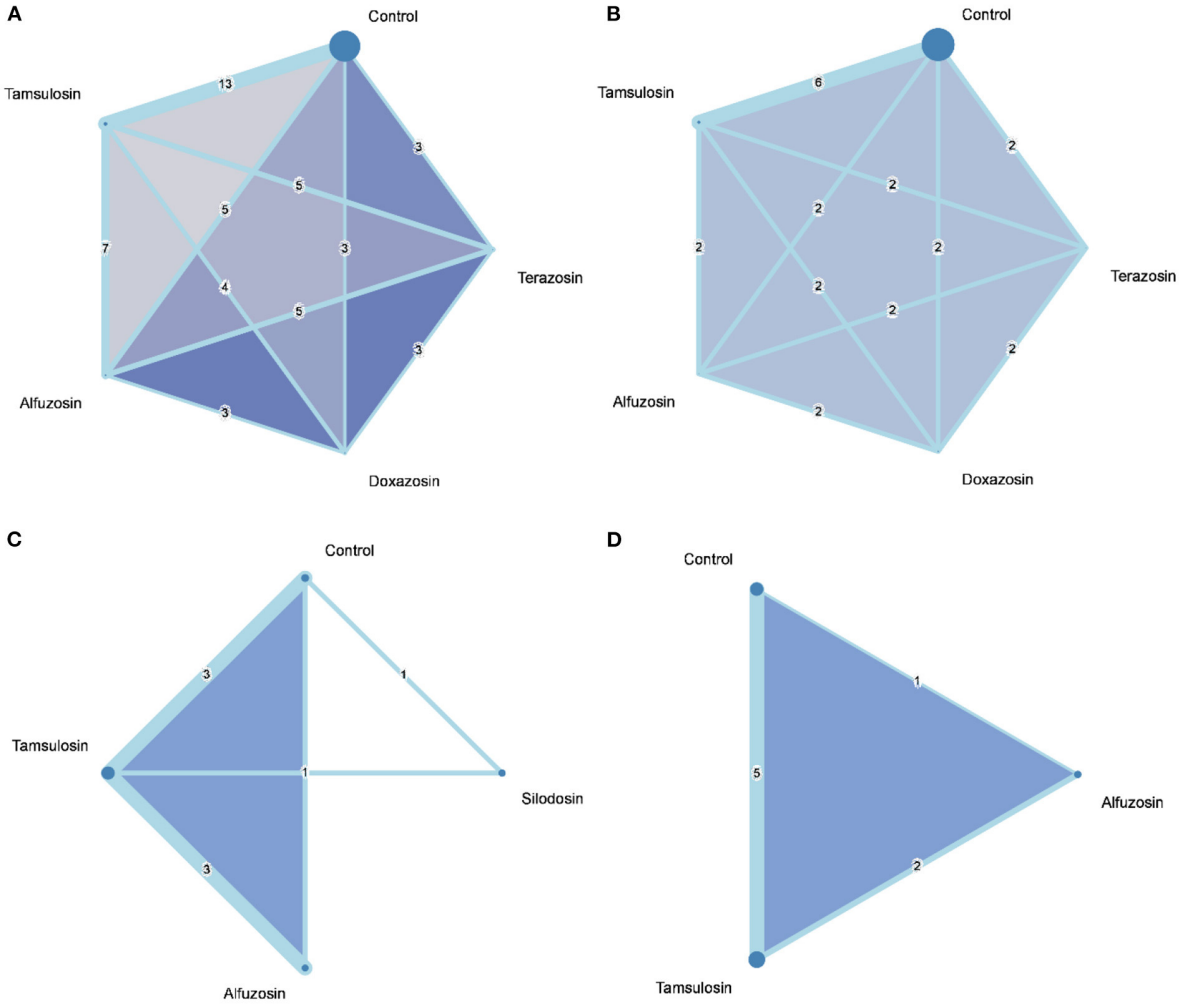
Network geometry of the consistency models for **(A)** intraoperative floppy iris syndrome, **(B)** severe intraoperative floppy iris syndrome, **(C)** mesopic pupil diameter, **(D)** dilated pupil diameter.

**Figure 3:**
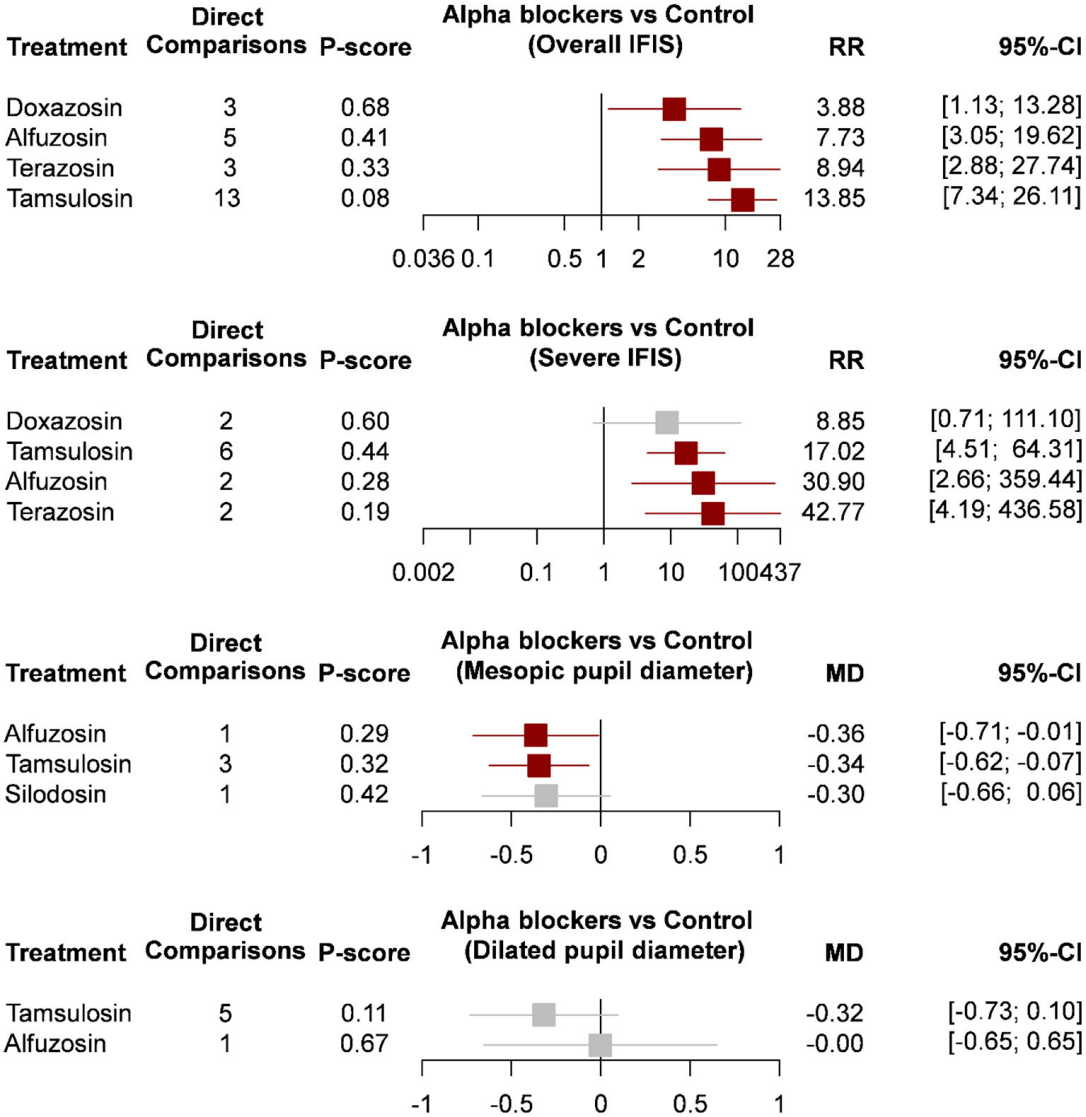
Forest plots of intraoperative floppy iris syndrome and pupil diameter. CI, confidence interval; IFIS, intraoperative floppy iris syndrome; MD, mean difference; RR, risk ratio.

Data on overall IFIS incidence were presented in 15 studies (Figure 2A) (20–34, 38, 39), and severe IFIS incidence was available in six studies (Figure 2B) (20, 21, 25, 28, 33, 34). Based on 51 direct comparisons with 6,488 cases, the consistency model of overall IFIS incidence consisted of five nodes, including control, tamsulosin, alfuzosin, doxazosin, and terazosin. The pooled results of direct evidence regarding overall IFIS incidence exhibited heterogeneity (Supplementary Figure S1), particularly within the comparison of tamsulosin and control (I-square = 93%; *P*-value < 0.01), as well as comparison between terazosin and control (I-square = 70%; *P*-value = 0.04). However, the statistical heterogeneity might be acceptable due to similar findings regarding overall IFIS incidence in tamsulosin and terazosin groups over the control group among all studies. Although no significant differences in IFIS incidence among the three selective α1-blockers, the consistency model showed that a higher risk of IFIS in tamsulosin (RR = 13.85; 95% CI: 7.34 to 26.11), terazosin (RR, 8.94; 95% CI 2.88 to 27.74), alfuzosin (RR = 7.73; 95% CI: 3.05 to 19.62), and doxazosin (RR = 3.88; 95% CI: 1.13 to 13.28) groups, as compared with the control group. Moreover, overall IFIS incidence in the doxazosin group was significantly lower than in the tamsulosin group (RR, 0.28; 95% CI 0.09 to 0.91). A similar trend is apparent in the *P*-scores (Figure 3A).

Based on six studies with 2,460 cases, a five-node consistency model examined the risk of severe IFIS among control tamsulosin, alfuzosin, doxazosin, and terazosin groups. The pooled results of direct evidence on severe IFIS incidence were also heterogeneous (Supplementary Figure S2), and statistical heterogeneity existed in the comparison of tamsulosin and doxazosin (I-square = 83%; *P*-value = 0.02). In the consistency model, the risk of severe IFIS in tamsulosin (RR = 17.02; 95% CI: 4.51 to 64.31), alfuzosin (RR = 30.90; 95% CI: 2.66 to 359.44), and terazosin (RR, 42.77; 95% CI 4.19 to 436.58) groups were significantly higher than in control group. A similar trend is apparent in the *P*-scores (Figure 3B). Although the risks of severe IFIS in the doxazosin group did not reach statistical significance, the risk ratio was also very high. No significant difference in the risk of severe IFIS was observed among the four selective α1-blockers.

### Pupil diameter

A total of 11 comparisons among seven studies (*n* = 564) contributed to a four-node consistency model of pupil diameter under mesopic light level (12, 13, 19, 20, 25, 40, 41), and six studies (*n* = 400) with eight comparisons formed a three-node consistency model of pupil diameter after dilation (12, 19, 20, 27, 34, 36). Regarding mesopic pupil diameter, statistical heterogeneity existed in the direct evidence of pairwise comparison between tamsulosin and alfuzosin (I-square = 78%; *P*-value < 0.01; Supplementary Figure S3), while the pooled estimate was not seriously affected by any single study after leave-one-out sensitivity analysis. The results of the network meta-analysis showed that tamsulosin (MD = −0.36; 95% CI: −0.71 to −0.01) and alfuzosin (MD = −0.34; 95% CI: −0.62 to −0.07) associated with smaller pupil diameter under mesopic light levels when control as the reference group. Pupil diameter in the silodosin group was not significantly smaller than in the control group, and no significant difference in mesopic pupil diameter among the three selective α1-blockers.

According to the data from eight direct comparisons, statistical heterogeneities were evidently observed in the direct evidence of dilated pupil diameter, particularly between the comparison of tamsulosin and alfuzosin groups (I-square = 83%; *P*-value = 0.02), as well as the pairwise comparison of tamsulosin and control groups (I-square = 76%; *P*-value < 0.01; Supplementary Figure S4). Based on the consistency model, no significant difference in dilated pupil diameter among the control, tamsulosin, and alfuzosin groups.

### Tests of incoherence and small-study effects

**Figure 4:**
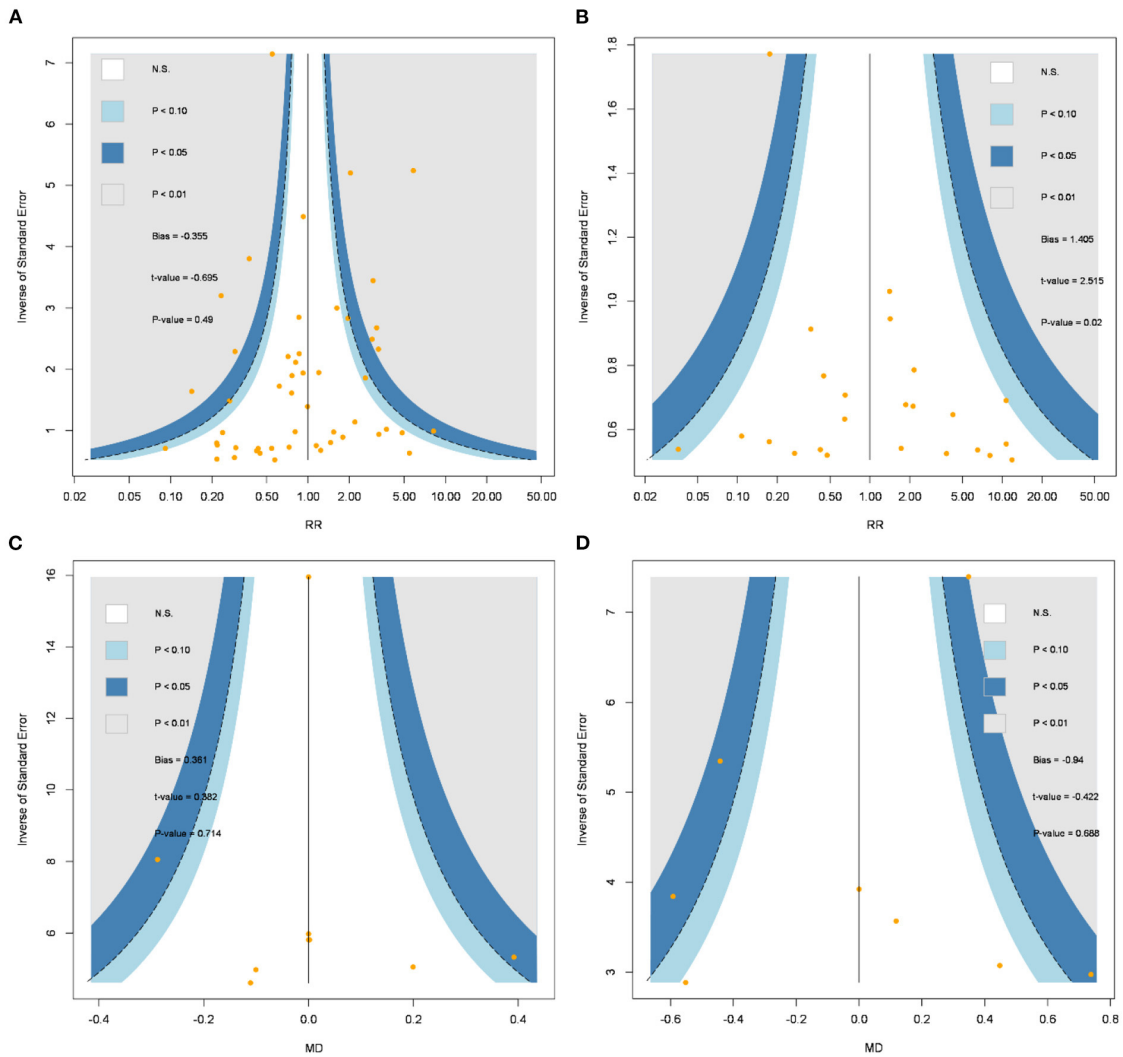
Comparison-adjusted funnel plots of **(A)** intraoperative floppy iris syndrome, **(B)** severe intraoperative floppy iris syndrome, **(C)** mesopic pupil diameter, **(D)** dilated pupil diameter. MD, mean difference; N.S., non-significance; RR, risk ratio.

Results of the incoherence tests were non-significant within all the network meta-analyses of overall IFIS (*Q* = 4.46; *P*-value = 0.985), severe IFIS (*Q* = 1.69; *P*-value = 0.194), mesopic pupil diameter (*Q* = 0.51; *P*-value = 0.916), and dilated pupil diameter (*Q* = 3.32; *P*-value = 0.191). Figure 4 presented comparison-adjusted funnel plots, and small-study effects appeared to not seriously affect pooled estimates in the network meta-analysis of overall IFIS (*t* = −0.70; *P*-value = 0.49), mesopic pupil diameter (*t* = 0.38; *P*-value = 0.714), and dilated pupil diameter (*t* = −0.42; *P*-value = 0.688). Small-study effect behind the pooled estimate of severe IFIS raised some concerns because of the significance of Egger's regression intercept test (*t* = 2.52; *P*-value = 0.02), but it might be not serious due to the symmetric pattern of comparison-adjusted funnel plot with non-significance after rank correlation test based on Begg's method (*z* = 1.07; *P*-value = 0.286).

## Discussion

### Key findings

This study observed that most α1-blockers are associated with a higher risk of IFIS with smaller pupil diameters under mesopic light levels. Among the usage of α1-antagonists, moreover, tamsulosin exhibits the highest RR and *P*-score. Though doxazosin is significantly associated with a higher risk of overall IFIS it does not significantly relate to the risk of severe IFIS. There is no available data on overall or severe IFIS incidence in the silodosin group although it does not significantly decrease mesopic pupil diameter. Unfortunately, there is also a paucity of evidence on the associations between IFIS and the other commonly used selective α1-blockers in terms of naftopidil.

Though there is no available human data yet; α1-A receptors have been shown to be the predominant subtype in the iris dilator muscle in most animal studies two decades ago (42, 43). Tamsulosin has the highest affinity to α1-A receptors and the highest IFIS incidence among the four regimens in the current study. Hypertension has also been demonstrated as an independent risk factor for IFIS (38, 44, 45). It is unclear whether this risk is associated with antihypertensive medications, or hypertension itself on iris physiology (46). While 2017 Guideline for High Blood Pressure in Adults state that doxazosin can be used as monotherapy for hypertension in a patient with LUTS or BPH (47). The effect of less selectivity to α1-A receptors, which low blood pressure, might give doxazosin the power in IFIS protection. Despite of the lowest IFIS incidence, it might be severe when occurring; we could not process a reasonable derivation with current data; exposure time (medicated duration) seemed not an independent factor for IFIS incidence or severity.

Silodosin, as a newer uroselective α1-antagonist, has demonstrated the highest selectivity for the α1a-AR subtype, with 583-fold and 56-fold higher binding affinity compared with the α1b and α1d subtypes, respectively (48). We found three case reports describing IFIS with patients taking silodosin (49–51). While naftopidil may pharmacologically induce less IFIS, naftopidil is the only compound developed with a distinct selectivity for the α1d-AR subtype (52), which we could not find any documented IFIS with naftopidil. Further studies are needed to address alpha adrenergic receptor subtypes in the human iris, the detailly pharmacokinetics that alpha blockers affect pupil, and cataract surgery.

The mean age of included studies ranged from 58 ± 4.3 to 79.9 ± 7.3 years, this did not decline the clinical applicability of the current article; BPH and PCS both shared a wide age range (14, 15). Other potential risk factors of IFIS might be controlled in different settings among included studies, which included but are not limited to age, gender, race, the axial length of the eye, ocular comorbidities such as pseudoexfoliation syndrome and glaucoma, hypertension, diabetes mellitus, 5α-reductase inhibitors, other α adrenoceptor antagonists or neuromodulators (13, 16, 38, 49, 53, 54).

Routinely perioperative preparation was in different settings among the included studies, such as different concentrations of topical mydriatics and phenylephrine given before surgery, adding topical non-steroidal anti-inflammatory drugs or not, the mixture of different concentrations of phenylephrine and ketorolac added into irrigation solution during surgery, with or without intracameral injection of mydriatics; some didn't state their preparation in detail. Different perioperative preparation should influence the occurrence of IFIS, add heterogeneity in our meta-analysis, and even make our result underestimated.

Indirect comparisons and limited direct comparisons between α1-antagonists demonstrate that all α1-antagonists have similar efficacy in appropriate doses (55). Patients usually took the same α1-antagonist for BPH/LUTS stably and regularly, some patients might experience medication adjustment, mostly due to complications [S. (56)]. Medication adjustment might reinforce or weaken the clinical applicability of the current article.

### Clinical implication

To prevent IFIS, which was associated with surgical complications, specific preoperative and intraoperative interventions should be considered. Cataract surgeons need not only to review the patient's medical history in detail but also to prepare yourselves to face IFIS and smaller dilated pupils, which is a restricted and unstable surgical field. A comprehensive medical history should be obtained and documented routinely before cataract surgery, caution should be paid in patients using α1-antagonists, especially Tamsulosin. Sex ratio varies in the includes studies, and the disproportionate ratio might affect the results due to the different conditions in the use of α1-antagonists. For instance, females might be prescribedα1-antagonists against urolithiasis, dysfunctional voiding, primary bladder neck obstruction, or other urological problems (57); we should assess the usage and consequence of α1-antagonists according to medical history instead of patients' gender.

Since nearly all male BPH/LUTS patients were candidates for cataract surgery and PCS, urologists and general physicians might consider doxazosin to be the first regimen, which resulted in less IFIS, if patients were hypertensive or could tolerate the adverse effects.

### Limitations

Although the present study provided informatic findings of IFIS and pupil diameter after selective α1 blocker in patients with BPH or LUTS by conducting network meta-analysis, original study designs and bias raise some concerns. This synthesis has the following limitations. Firstly, there are limited RCTs on this topic; wherefore transitivity would be violated. However, the present synthesis does not detect serious incoherence. For another thread, IFIS is more like an intraoperative complication rather than an orthodox disease, which was rationally related to surgeon's experience; included data on medication duration was scraggly and hard to collect due to different study setup processes or inclusion criteria. These potential biases seem to be non-differentiated issues, while their influences might not be ignored. Another limitation is that there is a wide range in the duration of the usage of selective α1 blocker among the included studies. The exposure time could not be well-controlled in this synthesis. Fourthly, our study identified five selective α1 blockers for patients with BPH or LUTS, but direct evidence on the selectiveα1 blocker (doxazosin) with the lower risk of IFIS only relied on a relatively small sample size (*n* = 102). Similarly, direct evidence on silodosin was only based on a limited sample size (*n* = 115). Besides, no evidence of naftopidil meets the eligibility criteria of this synthesis. Due to the limitations abovementioned, this topic still warrants more high-quality RCTs.

## Conclusions

IFIS seems inevitable with the usage of α1-antagonists, and tamsulosin needs to be cautious due to the significantly higher risk of IFIS; on the other hand, doxazosin is not significantly related to severe IFIS. Although silodosin does not significantly decrease mesopic pupil diameter, there is no conclusive evidence to support the recommendation of the use of silodosin due to insufficient evidence on the IFIS after silodosin. Optimized studies are craved to address their relationships with severe IFIS. Ophthalmologists, urologists, and physicians should be aware of these safety signals, especially in patients at high risk. Ophthalmologists should routinely review patients' medical history and prepare themselves to face an intraoperative emergency. Urologists and physicians may consider doxazosin as the first regimen, which resulted in less IFIS if patients are hypertensive or can tolerate the adverse effects.

## Data availability statement

The original contributions presented in the study are included in the article/Supplementary material, further inquiries can be directed to the corresponding author.

## Author contributions

Y-HW and L-CH: conceptualization, data curation, and writing—original draft. Y-HW, L-CH, and Y-JC: Investigation. ST and Y-NK: methodology and formal analysis. Y-JC and C-LW: Supervision. Y-NK: Visualization. ST, Y-JC, C-LW, and Y-NK: Writing—review and editing. All authors approved the final manuscript.

## Conflict of interest

The authors declare that the research was conducted in the absence of any commercial or financial relationships that could be construed as a potential conflict of interest.

## Publisher's note

All claims expressed in this article are solely those of the authors and do not necessarily represent those of their affiliated organizations, or those of the publisher, the editors and the reviewers. Any product that may be evaluated in this article, or claim that may be made by its manufacturer, is not guaranteed or endorsed by the publisher.

## Abbreviations

BPH: benign prostatic hyperplasia
CI: confidence interval
IFIS: intraoperative floppy iris syndrome
LUTS: lower urinary tract syndrome
MD: mean difference
PCS: phacoemulsification surgery
RR: risk ratio.
