# Supplementary material for: Risk of intraoperative floppy iris syndrome among selective alpha-1 blockers—A consistency model of 6,488 cases
Source: Front Med (Lausanne). 2022 Aug 30;9:941130. doi: 10.3389/fmed.2022.941130 (PMC9468244; doi:10.3389/fmed.2022.941130)

Frontiers in Medicine

Risk of Intraoperative Floppy Iris Syndrome among Selective Alpha-1 Blockers - A Consistency Model of 6488 Cases

Appendix

Ya-Hui Wang, MD[#1,2], **Liang-Chen Huang**, MD[#3], **Sung Huang Laurent Tsai**, MD MPH[4,5], **Ying-Jen Chen**, MD[5,6], **Chien-Liang Wu**, MD[1,2], **Yi-No Kang**, MA[*7,8,9,10,11]

1. Department of Ophthalmology, Taipei Municipal Wanfang Hospital, Taipei 116, Taiwan
2. School of Medicine, Taipei Medical University, Taipei 110, Taiwan
3. Division of Urology, Department of Surgery, En Chu Kong Hospital, New Taipei city 237, Taiwan
4. Department of Orthopaedic Surgery, Chang Gung Memorial Hospital, Keelung Branch, Keelung 204, Taiwan
5. School of Medicine, Chang Gung University, Taoyuan 333, Taiwan
6. Department of Geriatric and General Internal Medicine Chang Gung Memorial Hospital, Taoyuan 333, Taiwan
7. Evidence-Based Medicine Center, Wan Fang Hospital, Taipei Medical University, Taipei 116, Taiwan
8. Research Center of Big Data and Meta-analysis, Wan Fang Hospital, Taipei Medical University, Taipei, Taiwan
9. Cochrane Taiwan, Taipei Medical University, Taipei 110, Taiwan
10. Institute of Health Policy and Management, College of Public Health, National Taiwan University, Taipei 100, Taiwan.
11. Department of Health Care Management, College of Health Technology, National Taipei University of Nursing Health Sciences, Taipei, Taiwan

# Co-first: Ya-Hui Wang, MD and Liang-Chen Huang, MD contribute equally.
* Correspondence: Yi-No Kang, M.A., consultant

Table of Contents

Table S1. Databases and search strategy
Table S2. Risk of bias assessment
Figure S1. Forest plot of direct evidence on intraoperative floppy iris syndrome
Figure S2. Forest plot of direct evidence on severe intraoperative floppy iris syndrome
Figure S3. Forest plot of direct evidence on mesopic pupil diameter
Figure S4. Forest plot of direct evidence on dilated pupil diameter

Table S1 Primary search strategy

No.	Term and combinations
#01	Prostatic Hyperplasia
#02	urinary
#03	void
#04	#01 OR #02 OR #03
#05	Alpha Antagonist
#06	alpha blocker
#07	terazosin
#08	tamsulosin
#09	alfuzosin
#10	silodosin
#11	naftopidil
#12	doxazosin
#13	#05 OR #06 OR #07 #08 OR #08 OR #10 OR #11 OR #12
#14	Cataract
#15	Phacoemulsification
#16	floppy iris
#17	Lens
#18	Iris
#19	Cornea
#20	Choroid
#21	Pupil
#22	Ophthalmology
#23	#14 OR #15 OR #16 OR #17 OR #18 OR #19 OR #20 OR #21 OR #22
#24	#4 AND #13 AND #23

Table S1 Database and search strategy

Database	Search strategy	Returns
Cochrane	(Prostatic Hyperplasia OR urinary OR void) AND (Alpha Antagonist OR alpha blocker OR terazosin OR tamsulosin OR alfuzosin OR silodosin OR naftopidil OR doxazosin) AND (Cataract OR Phacoemulsification OR floppy iris OR Lens OR Iris OR Cornea OR Choroid OR Pupil OR Ophthalmology)	36
Embase	('bph (benign prostatic hyperplasia)'/exp OR 'bph (benign prostatic hyperplasia)' OR 'adenofibromyomatosis'/exp OR 'adenofibromyomatosis' OR 'benign hyperplasia, prostate'/exp OR 'benign hyperplasia, prostate' OR 'benign hyperplasia, prostatic'/exp OR 'benign hyperplasia, prostatic' OR 'benign hypertrophy, prostate'/exp OR 'benign hypertrophy, prostate' OR 'benign hypertrophy, prostatic'/exp OR 'benign hypertrophy, prostatic' OR 'benign prostate hyperplasia'/exp OR 'benign prostate hyperplasia' OR 'benign prostatic hyperplasia'/exp OR 'benign prostatic hyperplasia' OR 'benign prostatic hypertrophy'/exp OR 'benign prostatic hypertrophy' OR 'hyperplasia, prostate'/exp OR 'hyperplasia, prostate' OR 'hypertrophy, prostate'/exp OR 'hypertrophy, prostate' OR 'prostate benign hyperplasia'/exp OR 'prostate benign hyperplasia' OR 'prostate benign hypertrophy'/exp OR 'prostate benign hypertrophy' OR 'prostate enlargement'/exp OR 'prostate enlargement' OR 'prostate gland hypertrophy'/exp OR 'prostate gland hypertrophy' OR 'prostate hyperplasia'/exp OR 'prostate hyperplasia' OR 'prostate hyperthrophy'/exp OR 'prostate hyperthrophy' OR 'prostate hypertrophia'/exp OR 'prostate hypertrophia' OR 'prostate hypertrophy'/exp OR 'prostate hypertrophy' OR 'prostatic benign hyperplasia'/exp OR 'prostatic benign hyperplasia' OR 'prostatic benign hypertrophy'/exp OR 'prostatic benign hypertrophy' OR 'prostatic hyperplasia'/exp OR 'prostatic hyperplasia' OR 'prostatic hypertrophy'/exp OR 'prostatic hypertrophy') AND ('adrenergic alpha antagonists'/exp OR 'adrenergic alpha antagonists' OR 'adrenergic alpha-antagonists'/exp OR 'adrenergic alpha-antagonists' OR 'alpha adrenergic antagonist'/exp OR 'alpha adrenergic antagonist' OR 'alpha adrenergic blocker'/exp OR 'alpha adrenergic blocker' OR 'alpha adrenergic blocking agent'/exp OR 'alpha adrenergic blocking agent' OR 'alpha adrenergic blocking drug'/exp OR 'alpha adrenergic blocking drug' OR 'alpha adrenergic receptor antagonist'/exp OR 'alpha adrenergic receptor antagonist' OR 'alpha adrenergic receptor blocker'/exp OR 'alpha adrenergic receptor blocker' OR 'alpha adrenergic receptor blocking agent'/exp OR 'alpha adrenergic receptor blocking agent' OR 'alpha adrenoceptor antagonist'/exp OR 'alpha adrenoceptor antagonist' OR 'alpha adrenoceptor blocker'/exp OR 'alpha adrenoceptor blocker' OR 'alpha adrenoceptor blocking agent'/exp OR 'alpha adrenoceptor blocking agent' OR 'alpha adrenoceptor blocking drug'/exp OR 'alpha adrenoceptor blocking drug' OR 'alpha adrenolytic agent'/exp OR 'alpha adrenolytic agent' OR 'alpha antiadrenergic agent'/exp OR 'alpha antiadrenergic agent' OR 'alpha blocker'/exp OR 'alpha blocker' OR 'alpha blocking agent'/exp OR 'alpha blocking agent' OR 'alpha receptor blocker'/exp OR 'alpha receptor blocker' OR 'alpha receptor blocking agent'/exp OR 'alpha receptor blocking agent' OR 'alpha sympathicolytic agent'/exp OR 'alpha sympathicolytic agent' OR 'antiadrenergics, alpha blocking'/exp OR 'antiadrenergics, alpha blocking') AND ('eye pupil'/exp OR 'eye pupil' OR 'pupil'/exp OR 'pupil' OR 'pupil diameter'/exp OR 'pupil diameter' OR 'pupil size'/exp OR 'pupil size' OR 'pupilla'/exp OR 'pupilla' OR 'iris'/exp OR 'iris' OR 'choriopathy'/exp OR 'choriopathy' OR 'choroid disease'/exp OR 'choroid disease' OR 'choroid diseases'/exp OR 'choroid diseases' OR 'choroid disorder'/exp OR 'choroid disorder' OR 'choroidal lesion'/exp OR 'choroidal lesion' OR 'choroidopathy'/exp OR 'choroidopathy' OR 'floppy iris syndrome'/exp OR 'floppy iris syndrome' OR 'cataract'/exp OR 'cataract' OR 'cataract, lens'/exp OR 'cataract, lens' OR 'cataract, lenticular'/exp OR 'cataract, lenticular' OR 'cataract, total'/exp OR 'cataract, total' OR 'cataractous lens'/exp OR 'cataractous lens' OR 'eye lens opacity'/exp OR 'eye lens opacity' OR 'lens cataract'/exp OR 'lens cataract' OR 'lens clouding'/exp OR 'lens clouding' OR 'lens opacity'/exp OR 'lens opacity' OR 'lenticular cataract'/exp OR 'lenticular cataract' OR 'nuclear cataract'/exp OR 'nuclear cataract' OR 'opacification, posterior capsule'/exp OR 'opacification, posterior capsule' OR 'opacity, lens'/exp OR 'opacity, lens' OR 'posterior capsule opacification'/exp OR 'posterior capsule opacification' OR 'total cataract'/exp OR 'total cataract' OR 'cornea'/exp OR 'cornea' OR 'cornea chemistry'/exp OR 'cornea chemistry' OR 'cornea development'/exp OR 'cornea development' OR 'cornea diameter'/exp OR 'cornea diameter' OR 'cornea sensation'/exp OR 'cornea sensation' OR 'cornea structure'/exp OR 'cornea structure' OR 'crystalline lens'/exp OR 'crystalline lens' OR 'eye lens'/exp OR 'eye lens' OR 'eyelens'/exp OR 'eyelens' OR 'lens'/exp OR 'lens' OR 'lens substance'/exp OR 'lens substance' OR 'lens, crystalline'/exp OR 'lens, crystalline' OR 'lenses'/exp OR 'lenses' OR 'substantia lentis'/exp OR 'substantia lentis' OR 'ophthalmologic examination'/exp OR 'ophthalmologic examination' OR 'ophthalmology'/exp OR 'ophthalmology' OR 'space ophthalmology'/exp OR 'space ophthalmology')	145

Table S1 (continue) Database and search strategy

Database	Search strategy	Returns
PubMed	Query<br>(Prostatic Hyperplasia OR urinary OR void) AND (Alpha Antagonist OR alpha blocker OR terazosin OR tamsulosin OR alfuzosin OR silodosin OR naftopidil OR doxazosin) AND (Cataract OR Phacoemulsification OR floppy iris OR Lens OR Iris OR Cornea OR Choroid OR Pupil OR Ophthalmology)<br><br>Search details:<br>("prostatic hyperplasia"[MeSH Terms] OR ("prostatic"[All Fields] AND "hyperplasia"[All Fields]) OR "prostatic hyperplasia"[All Fields] OR ("urinary tract"[MeSH Terms] OR ("urinary"[All Fields] AND "tract"[All Fields]) OR "urinary tract"[All Fields] OR "urinary"[All Fields]) OR ("urination"[MeSH Terms] OR "urination"[All Fields] OR "void"[All Fields])) AND ((("alpha"[All Fields] OR "alpha s"[All Fields] OR "alphas"[All Fields]) AND ("antagonist"[All Fields] OR "antagonists and inhibitors"[MeSH Subheading] OR ("antagonists"[All Fields] AND "inhibitors"[All Fields]) OR "antagonists and inhibitors"[All Fields] OR "antagonists"[All Fields])) OR ("adrenergic alpha antagonists"[Pharmacological Action] OR "adrenergic alpha antagonists"[MeSH Terms] OR ("adrenergic"[All Fields] AND "alpha antagonists"[All Fields]) OR "adrenergic alpha antagonists"[All Fields] OR ("alpha"[All Fields] AND "blocker"[All Fields]) OR "alpha blocker"[All Fields]) OR ("terazosin"[Supplementary Concept] OR "terazosin"[All Fields] OR "terazosin"[All Fields] OR "terazosine"[All Fields]) OR ("tamsulosin"[MeSH Terms] OR "tamsulosin"[All Fields] OR "tamsulosine"[All Fields]) OR ("alfuzosin"[Supplementary Concept] OR "alfuzosin"[All Fields]) OR ("silodosin"[Supplementary Concept] OR "silodosin"[All Fields] OR "silodosin s"[All Fields]) OR ("naftopidil"[Supplementary Concept] OR "naftopidil"[All Fields]) OR ("doxazosin"[MeSH Terms] OR "doxazosin"[All Fields] OR "doxazosine"[All Fields])) AND ("cataract"[MeSH Terms] OR "cataract"[All Fields] OR "cataracts"[All Fields] OR "cataractic"[All Fields] OR "cataractous"[All Fields] OR ("phacoemulsification"[MeSH Terms] OR "phacoemulsification"[All Fields] OR "phacoemulsifications"[All Fields] OR "phakoemulsification"[All Fields]) OR (("floppiness"[All Fields] OR "floppy"[All Fields]) AND ("iris"[MeSH Terms] OR "iris"[All Fields])) OR ("lenses"[MeSH Terms] OR "lenses"[All Fields] OR "lens"[All Fields] OR "lens, crystalline"[MeSH Terms] OR ("lens"[All Fields] AND "crystalline"[All Fields]) OR "crystalline lens"[All Fields]) OR ("iris"[MeSH Terms] OR "iris"[All Fields]) OR ("cornea"[MeSH Terms] OR "cornea"[All Fields] OR "corneas"[All Fields] OR "cornea s"[All Fields] OR "corneae"[All Fields]) OR ("choroid"[MeSH Terms] OR "choroid"[All Fields] OR "choroids"[All Fields] OR "choroidal"[All Fields] OR "choroidally"[All Fields] OR "choroidals"[All Fields] OR "choroideal"[All Fields] OR "choroiditis"[MeSH Terms] OR "choroiditis"[All Fields] OR "choroiditides"[All Fields]) OR ("pupil"[MeSH Terms] OR "pupil"[All Fields] OR "pupils"[All Fields] OR "pupil s"[All Fields]) OR ("ophthalmologie"[All Fields] OR "ophthalmology"[MeSH Terms] OR "ophthalmology"[All Fields] OR "ophthalmology s"[All Fields]))	147
Web of Science	Prostatic Hyperplasia OR urinary OR void (All Fields) and Alpha Antagonist OR alpha blocker OR terazosin OR tamsulosin OR alfuzosin OR silodosin OR naftopidil OR doxazosin (All Fields) and Cataract OR Phacoemulsification OR floppy iris OR Lens OR Iris OR Cornea OR Choroid OR Pupil OR Ophthalmology (All Fields)	59

Table S2 Quality Assessment of the Included Studies Using the Newcastle-Ottawa Scale

Author	Selection 1	Selection 2	Selection 3	Selection 4	Comparability 1	Comparability 2	Outcome 1	Outcome 2	Outcome 3	Total Quality Score
Aktas 2014	*	*	*	*	*		*		*	7 (good quality)
Altan 2007	*	*	*	*	*		*	*	*	8 (good quality)
Bidaguren 2007	*	*	*		*		*		*	6 (fair quality)
Casuccio 2011	*	*	*	*	*		*		*	7 (good quality)
Chadha 2007	*	*	*	*			*	*	*	7 (poor quality due to no comparability)
Chang 2014	*	*	*	*	*	*	*	*	*	9 (good quality)
Chatziralli 2016	*	*	*	*			*	*	*	7 (poor quality due to no comparability)
Dogan 2017	*	*	*	*	*	*	*	*	*	9 (good quality)
Goyal 2014	*	*	*	*			*	*	*	7 (poor quality due to no comparability)
Hargitai 2013	*		*	*	*	*	*	*	*	8 (good quality)
Hillelsohn 2015	*	*	*	*	*		*	*	*	8 (good quality)
Horvath 2011	*	*	*	*			*	*	*	7 (poor quality due to no comparability)
Kaczmarek 2017	*	*	*	*	*		*	*	*	8 (good quality)
Kanar 2021	*	*	*	*	*		*	*	*	8 (good quality)
Karaca 2021	*		*	*	*		*		*	6 (fair quality)
Keklikci 2009	*	*	*	*			*	*	*	7 (poor quality due to no comparability)
Klysik 2014	*	*	*	*	*		*	*	*	8 (good quality)
Lim 2014	*		*	*	*		*	*	*	7 (good quality)
Ozer 2013	*	*	*	*			*	*	*	7 (poor quality due to no comparability)

Table S2 (continue) Quality Assessment of the Included Studies Using the Newcastle-Ottawa Scale

**Author**	**Selection 1**	**Selection 2**	**Selection 3**	**Selection 4**	**Comparability 1**	**Comparability 2**	**Outcome 1**	**Outcome 2**	**Outcome 3**	**Total Quality Score**
Prata 2009	*		*	*	*		*		*	6 (fair quality)
Paulsen 2014	*	*	*	*	*		*	*	*	8 (good quality)
Takmaz 2007	*	*		*			*	*	*	6 (poor quality due to no comparability)
Panagiotis 2012	*		*	*	*		*		*	6 (fair quality)
Tufan 2013	*	*	*	*	*		*	*	*	8 (good quality)
Yuksel 2015		*	*	*	*		*		*	6 (fair quality)

Selection 1: Representativeness of Exposed Cohort
Selection 2: Selection of Non-Exposed Cohort
Selection 3: Ascertainment of Exposure
Selection 4: Demonstration That Outcome of Interest Was Not Present at Start of Study
Comparison 1: Adjust for the most important risk factors
Comparison 2: Adjust for other risk factors
Outcome 1: Assessment of outcome
Outcome 2: Follow-up length
Outcome 3: Loss to follow-up rate

Figure S1 Forest plot of direct evidence on intraoperative floppy iris syndrome

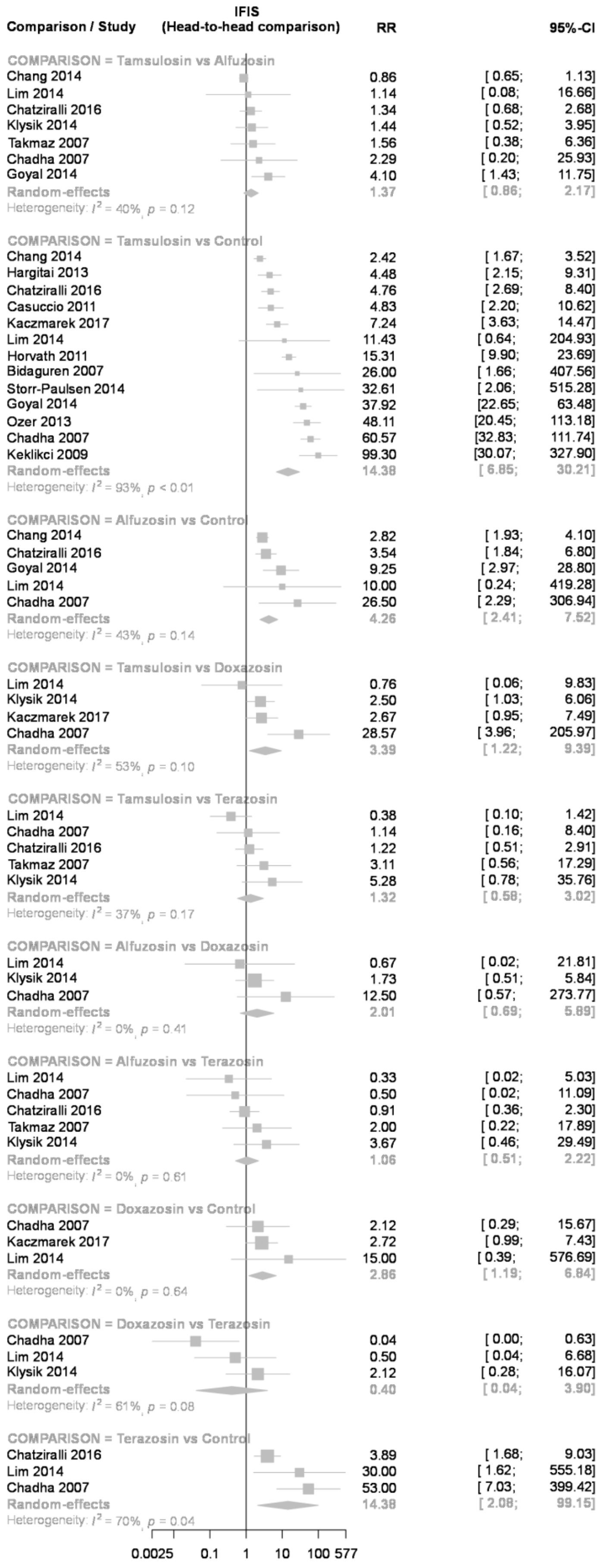

Figure S2 Forest plot of direct evidence on severe intraoperative floppy iris syndrome

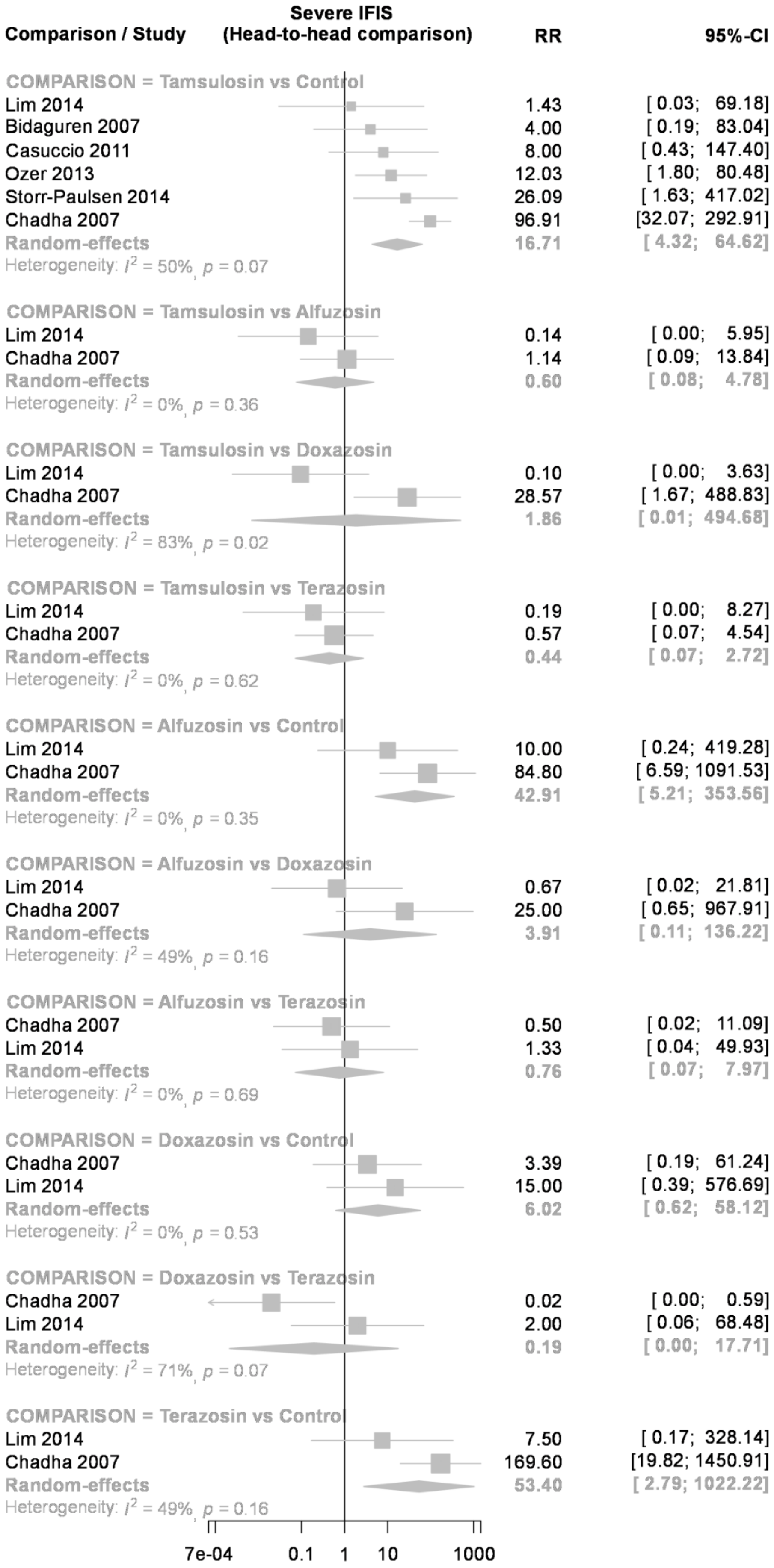

Figure S3 Forest plot of direct evidence on mesopic pupil diameter

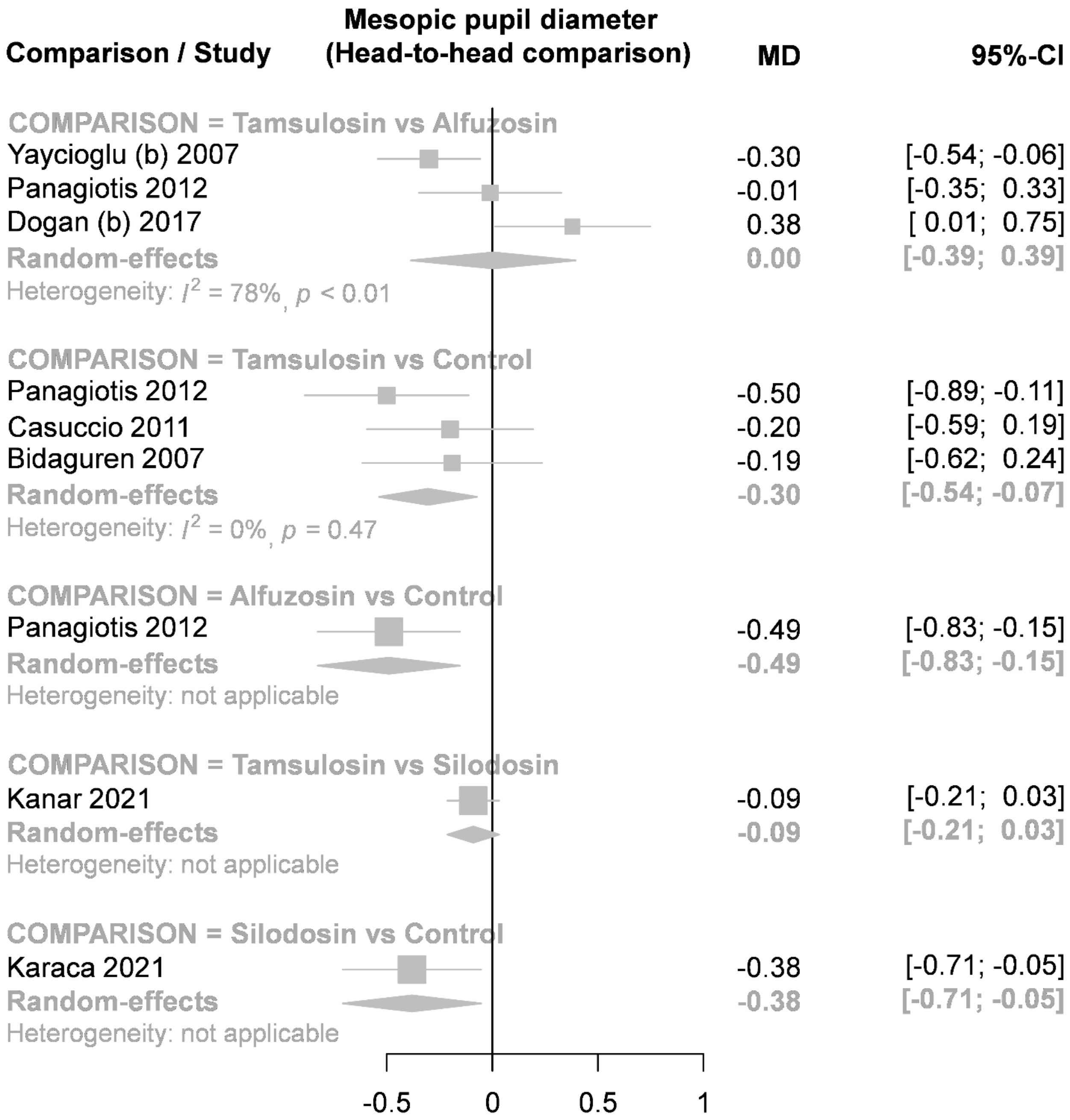

Figure S4 Forest plot of direct evidence on dilated pupil diameter

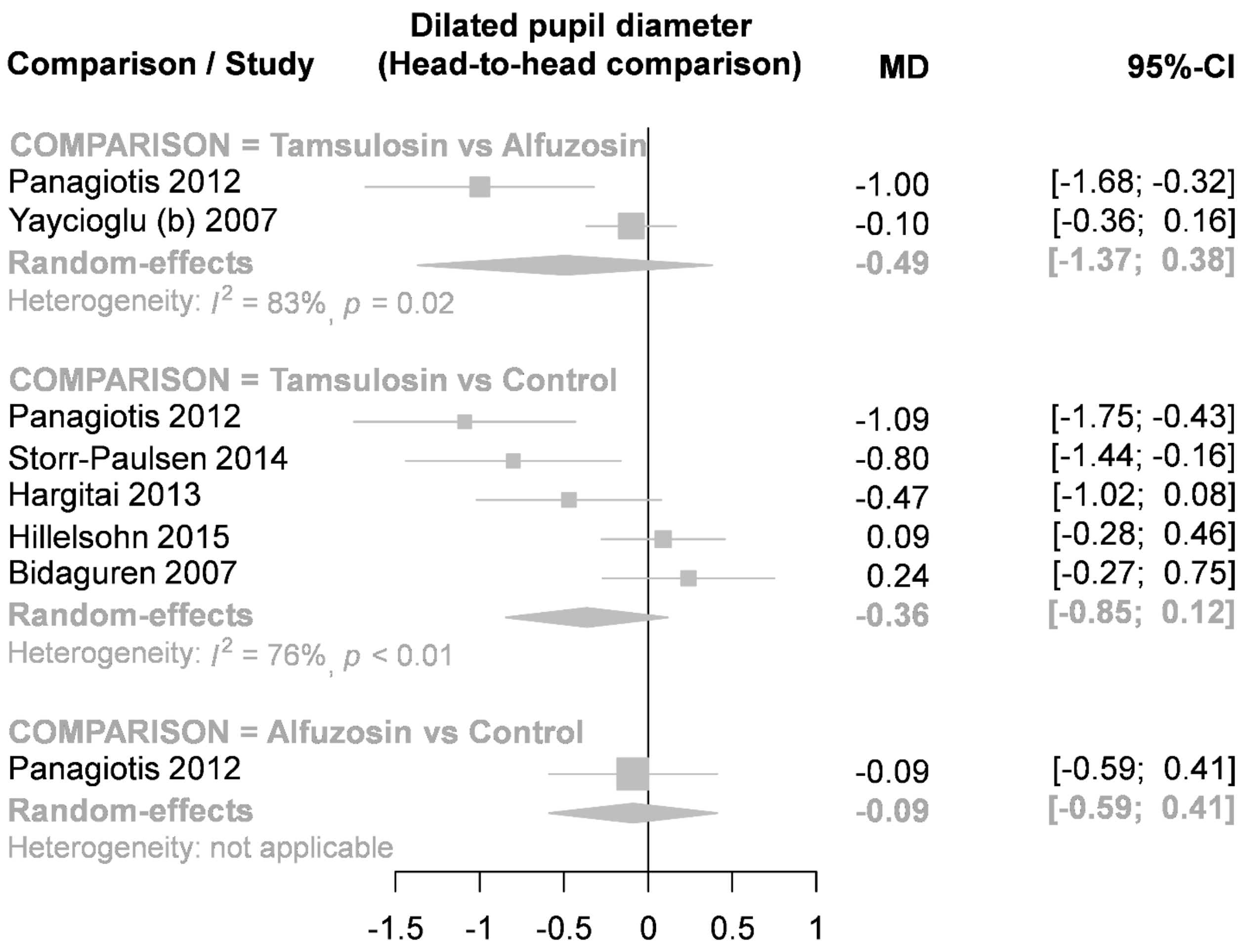

Supplement: Supplementary file 1 [file Presentation_1.pdf]
